# An Integrated Cytoskeletal Model of Neurite Outgrowth

**DOI:** 10.3389/fncel.2018.00447

**Published:** 2018-11-26

**Authors:** Kyle E. Miller, Daniel M. Suter

**Affiliations:** ^1^Department of Integrative Biology, Michigan State University, East Lansing, MI, United States; ^2^Department of Biological Sciences, Purdue University, West Lafayette, IN, United States; ^3^Purdue Institute for Integrative Neuroscience, Purdue University, West Lafayette, IN, United States; ^4^Bindley Bioscience Center, Purdue University, West Lafayette, IN, United States; ^5^Birck Nanotechnology Center, Purdue University, West Lafayette, IN, United States

**Keywords:** active matter, actin, axonal elongation, axonal transport, dynein, growth cone, microtubule, non-muscle myosin II

## Abstract

Neurite outgrowth underlies the wiring of the nervous system during development and regeneration. Despite a significant body of research, the underlying cytoskeletal mechanics of growth and guidance are not fully understood, and the relative contributions of individual cytoskeletal processes to neurite growth are controversial. Here, we review the structural organization and biophysical properties of neurons to make a semi-quantitative comparison of the relative contributions of different processes to neurite growth. From this, we develop the idea that neurons are active fluids, which generate strong contractile forces in the growth cone and weaker contractile forces along the axon. As a result of subcellular gradients in forces and material properties, actin flows rapidly rearward in the growth cone periphery, and microtubules flow forward in bulk along the axon. With this framework, an integrated model of neurite outgrowth is proposed that hopefully will guide new approaches to stimulate neuronal growth.

## Introduction

Neurite outgrowth is essential for wiring the nervous system during development and regeneration following trauma or disease (Suter and Miller, 2011; Kulkarni and Firestein, 2012; Budday et al., 2014; Hilton and Bradke, 2017; Stoeckli, 2018). Despite a significant body of research conducted over the last three decades, the underlying mechanisms of axonal growth and guidance are not fully understood, especially at the interface of dynamics and mechanics (Suter and Miller, 2011; Leterrier et al., 2017). Quoting Benford’s Law of Controversy, “Passion is inversely proportional to the amount of real information available” (Benford, 1980), we are excited to bring together quantitative data acquired by many labs to develop a mechanical model of neurite growth. The strength of this approach is that because forces can be mathematically integrated, the relative contributions of different processes can be impartially considered in a single framework. We begin this review by discussing the structural organization of neurons focusing on the actin and MT cytoskeleton. We then briefly summarize recent studies on the biophysics of growth cones and axons and use this perspective to discuss the cell biology of actin and MT dynamics, cellular adhesions, molecular force generation, and cytoskeletal cross-linkers. In the last sections, we discuss some of the “controversial” findings in this field in light of a more integrated model of neurite growth. Throughout this review, we develop the idea that neurons are active fluids, which generate strong contractile forces in the growth cone and weaker contractile forces along the axon through multiple interacting processes (O’Toole et al., 2015; de Rooij et al., 2018). As a result of subcellular gradients in forces, viscosity, and substrate adhesions, actin flows rapidly rearward in the growth cone periphery, and MTs flow forward in bulk along the axon (Athamneh et al., 2017). From this, a picture emerges that a growth cone is much like a migrating cell coupled to the cell body by the axon, much like “a leukocyte on a leash” (Pfenninger, 1986). Forces and cytoskeletal dynamics in the growth cone control its advance, while the axon acts to restrain and support growth cone motility. The significant difference of the present to previous models is that the growth cone advances as a coherent structure and pulls the adjacent axon forward, instead of elongating by the assembly of MTs at the tip of the axon (Dent and Gertler, 2003; Cammarata et al., 2016; Blanquie and Bradke, 2018). We conclude this review by touching on the implications of this updated model for developing approaches to promote rapid axonal elongation.

## Neuronal Structure

### The Structure of the Growth Cone

The highly motile structure at the tip of growing axons and dendrites is called the growth cone. Its key function in establishing the complex neuronal networks in the nervous system was recognized by Ramón y Cajal over 100 years ago. The two most critical cytoskeletal proteins involved in neurite outgrowth and guidance are actin filaments and MTs. Here, we briefly summarize their organization and dynamics including assembly, translocation, stabilization, and turnover to develop a foundation for understanding the mechanical process of neurite outgrowth. Several excellent reviews of these processes have been published recently (Coles and Bradke, 2015; Kapitein and Hoogenraad, 2015; Cammarata et al., 2016; Letourneau, 2016; Matamoros and Baas, 2016; Voelzmann et al., 2016; Omotade et al., 2017); therefore, we will not cover them in detail here.

**FIGURE 1:**
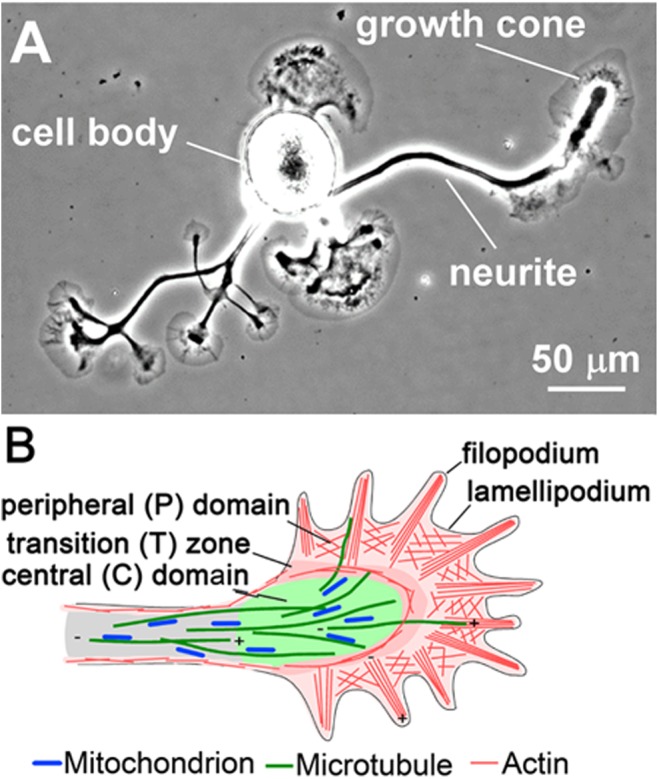
Overview of the neurite and growth cone. **(A)** Phase contrast image of an *Aplysia* bag cell neuron. **(B)** Schematic of the neuronal growth cone depicting different cytoplasmic regions and cytoskeletal structures. Adapted from O’Toole et al. (2015) with permission from Elsevier.

The growth cone is usually divided into three morphologically and functionally distinct cytoplasmic regions: (1) the peripheral domain, which is made up by filopodia and intervening lamellipodial veils; (2) the transition zone; and (3) the central domain, which is rich in various organelles including mitochondria (Figure 1). F-actin is the predominant cytoskeletal structure in the peripheral domain and transition zone, and at least four different subpopulations of F-actin structures are recognized in the growth cone. In the periphery, polarized bundles of 15–20 actin filaments provide the core structure of filopodia (Figure 1B), fingerlike protrusions that dynamically explore the environment for guidance information (Lewis and Bridgman, 1992; Davenport et al., 1993; Korobova and Svitkina, 2008; Gallo, 2013). The lamellipodia (Figure 1B) between the filopodia are filled with a dense, branched F-actin network, whose constant turnover drives the forward movement of the growth cone (Lewis and Bridgman, 1992; Small et al., 2002; Mongiu et al., 2007). The third subtype of F-actin structures are the transverse actin arcs that surround the central domain and control its shape as well as the distribution of MTs (Schaefer et al., 2002, 2008; Zhang et al., 2003). Lastly, the fourth F-actin structure is the dynamically rearranging intrapodia or ruffles in the transition zone (Rochlin et al., 1999), which recently have been suggested to promote traction force generation by buffering developing adhesion sites from the effects of retrograde flow (Buck et al., 2017).

**FIGURE 2:**
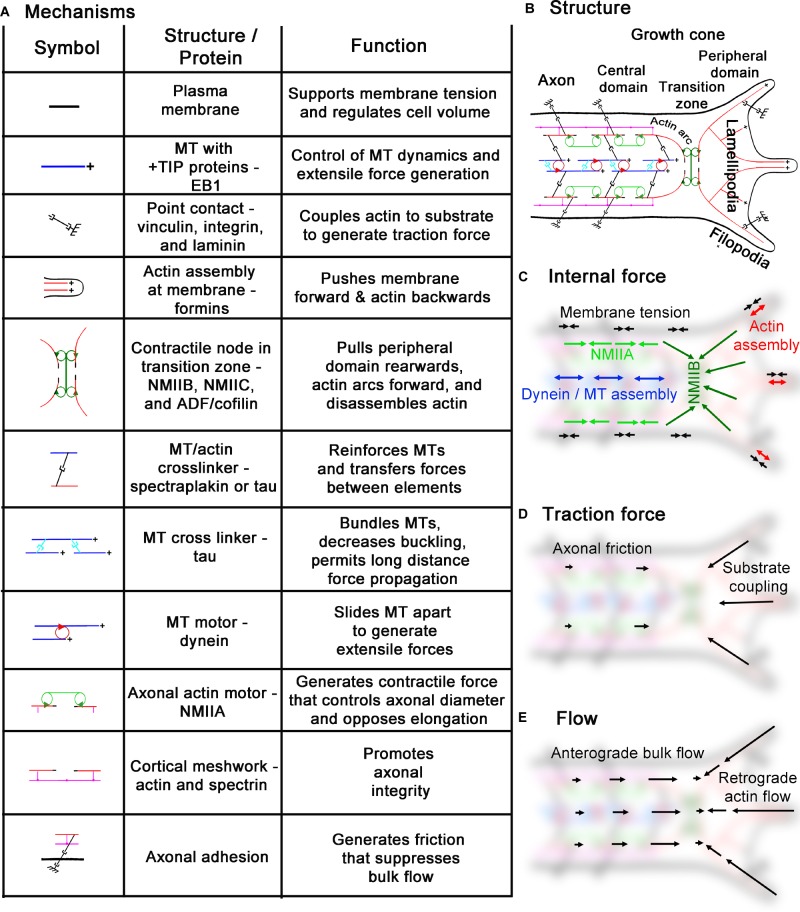
An integrated cytoskeletal model of neurite outgrowth. **(A)** Summary of the mechanisms, structures/proteins, and functions reviewed in the manuscript. **(B)** A diagram of the interrelationship between the structures. **(C)** Overview of significant sources of internal force generation; arrows pointing together indicate a contractile force dipole, a line with arrowheads on each end represents an extensile force dipole. The length of the arrows (or pairs of arrows) gives a relative indication of the force associated with each process. **(D)** Traction forces exerted on the substrate; the length of the arrows indicates relative magnitude. **(E)** Flow map, arrow length indicates relative velocity. The force and velocity vectors are shown over a blurred image of the underlying structure to give a sense of relative location.

The F-actin structures in the peripheral domain and transition zone are highly dynamic and turnover within a few minutes. Actin assembly occurs at the plus ends of filaments at filopodial tips and along the leading edge of lamellipodia to push the plasma membrane forward (Mallavarapu and Mitchison, 1999; Shahapure et al., 2010; Amin et al., 2012; Craig et al., 2012; Van Goor et al., 2012; Lee et al., 2013; Figure 2). Following assembly, F-actin moves by a process referred to as “retrograde actin flow,” which is mainly dependent on NMII (Medeiros et al., 2006). Lastly, actin filaments are disassembled in the transition zone by ADF/ cofilin (Marsick et al., 2010; Flynn et al., 2012; Omotade et al., 2017) and other proteins such as gelsolin (Lu et al., 1997). G-actin is transported to the leading edge to complete the cycle (Lee et al., 2013). As will be discussed below in more detail, a major function of these processes is to generate the forces needed for MT advance.

### The Structure of the Axon

#### Actin in the Axon

**FIGURE 3:**
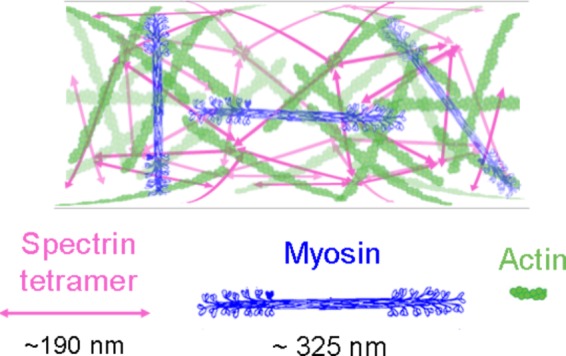
The axonal actin cortex as a weakly ordered meshwork. Hypothetical interactions of axonal NMII filaments with actin and spectrin in a weakly organized meshwork. Myosin filament reprinted from Niederman and Pollard (1975) with permission from Elsevier.

Whereas a significant body of literature has described the organization and dynamics of F-actin in the neuronal growth cone, less is known about the details of the F-actin cytoskeleton in the axon. Nonetheless, due to the recent developments in super-resolution microscopy, this is now rapidly changing with the recognition of actin rings, waves, trails, and patches (Roy, 2016; Leterrier et al., 2017; Papandreou and Leterrier, 2018). Of particular relevance to neuronal mechanics are actin ring structures in axons, which are capped at the plus ends by adducin and spaced at roughly 190 nm intervals by spectrin (Xu et al., 2013; Zhong et al., 2014; D’Este et al., 2015; Papandreou and Leterrier, 2018). While the function of the rings is still being determined, there are several lines of evidence suggesting that they play a key role in axonal mechanics along with the axonal actin cortex. In particular, spectrin is essential for maintaining the structural integrity of axons by resisting the stresses and strains arising from body motion (Hammarlund et al., 2007; Krieg et al., 2017). Likewise, NMII and adducin have an overlapping periodicity with the actin rings (Leite et al., 2016; Berger et al., 2018), and regulate axonal diameter (Leite et al., 2016; Fan et al., 2017). Since actin and NMII also drive axonal contraction and retraction (Joshi et al., 1985; Tofangchi et al., 2016), the actomyosin cortex appears to produce contractile forces both circumferentially and longitudinally along the length of the axons (Figure 3).

Whereas early models of actin rings proposed short filaments of ∼ 20 nm arranged in a circle (Xu et al., 2013), the length of these actin filaments (Jones and Svitkina, 2016) and their directionality remain unclear (Berger et al., 2018). In particular, electron microscopy has indicated that the cortical axonal actin is a random or weakly ordered meshwork of filaments with a length of roughly 1.5 μm (Hirokawa, 1982; Bearer and Reese, 1999; Leterrier et al., 2017). In addition, longer longitudinal actin structures with a mean length of 9 μm called trails (Ganguly et al., 2015) have been observed in many super-resolution studies (D’Este et al., 2015; Leite et al., 2016). Reconciling the observations of periodicity, a meshwork like organization, and NMII-driven contraction suggests the possibility that actin is a weakly ordered meshwork (Figure 3) that has periodic differences in density in mature axons that appear as rings. As NMII filaments are ∼ 300 nm long (Billington et al., 2013), they may wrap around the axon to generate a circumferential force (Berger et al., 2018), and span or lie diagonally between rings to generate a longitudinal force (de Rooij et al., 2018; Mutalik et al., 2018). In parallel, NMII filaments interconnecting trails (Ganguly et al., 2015) may generate forces that are propagated over long distances. This supports a speculative mechanistic hypothesis for the observations that axons, away from the growth cone, generate a net contractile force of ∼0.6 nN (O’Toole et al., 2015), NMII drives axonal contraction and retraction (Wylie and Chantler, 2003; Gallo, 2006; Myers et al., 2006; Brown et al., 2009; Tofangchi et al., 2016; Mutalik et al., 2018), and NMII generates contractile forces that control axonal diameter (Fan et al., 2017). As structure and function are intertwined, bridging the gap between the super-resolution imaging, electron microscopy, and biophysical studies seems likely to be a fruitful direction for investigations aimed at understanding neuronal mechanics.

#### MT Polarity and Length During Axonal Elongation

**FIGURE 4:**
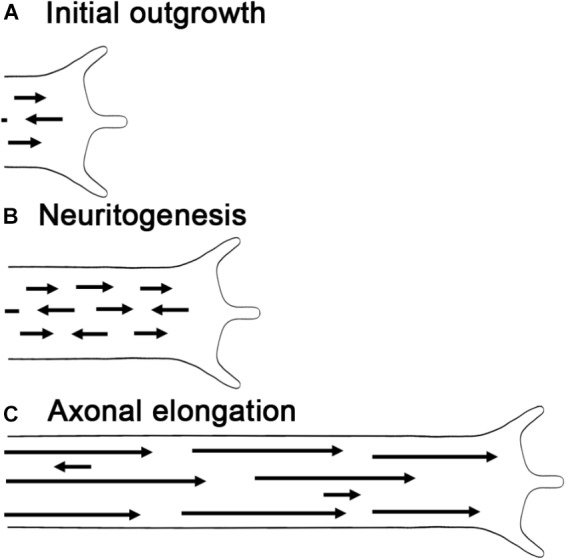
Microtubule polarity and length increase during axonal outgrowth. **(A)** Initial growth cone with the arrows representing the length and orientation of MTs. **(B)** During neurogenesis, MT sliding adds new short MTs with mixed orientations. **(C)** As axons elongate polarity and MT length increase, while sliding and MT number decrease.

In combination with F-actin, MTs are essential for axonal elongation and growth cone guidance (Coles and Bradke, 2015; Kapitein and Hoogenraad, 2015; Letourneau, 2016). Like actin filaments, MTs are polarized structures with polymerization occurring at their plus ends by the addition of tubulin dimers. In higher organisms, the majority of MTs have their plus ends oriented toward the axonal terminal, whereas dendrites exhibit a more mixed polarity (Baas et al., 1988; Yau et al., 2016). MT polarity is critical for the polarized organization of neurons, as it underlies the directional transport of proteins and organelles (Maday et al., 2014; Leterrier et al., 2017), the establishment of axon vs. dendrite identity (Rolls and Jegla, 2015), and the generation of forces through MT sliding (Jakobs et al., 2015; Kapitein and Hoogenraad, 2015; Kahn and Baas, 2016; de Rooij et al., 2017; Lu and Gelfand, 2017). As development progresses, MT polarity in neuronal processes becomes more ordered (van Beuningen et al., 2015). In lower organisms, such as *Drosophila* and *C. elegans*, dendritic MTs initially have a mixed polarity, which transitions to a nearly uniform minus ends out orientation (Maniar et al., 2011; Hill et al., 2012). Likewise in axons, the polarity of MTs increases over time. As an example in rat cortical neurons initially ∼80% of the MTs point toward the growth cone, but in mature axons nearly all do so (Baas et al., 1989; Yau et al., 2016; Figure 4).

In parallel to these changes in MT polarity, there are dramatic changes in the length and number of MT during development. Building on classic electron microscopy studies (Chalfie and Thomson, 1979; Bray and Bunge, 1981; Burton, 1987; Yu and Baas, 1994), a recent analysis in *C. elegans* provides new insights in how MTs are added to growing axons and its impact on organelle transport (Yogev et al., 2016). Using an innovative image analysis approach, the authors quantified the number, length and spacing of MTs in specific axons of in living worms. They achieved this by correlating the intensities of single MTs with the intensity of tubulin-GFP along the axon to determine the beginning and end of individual MTs. This approach has the advantages over electron microscopy in that data can be collected rapidly without time-consuming sample preparations and that MT dynamics can be observed directly. Examining the length and distribution of MTs from an early larval stage to adulthood, average MT length doubled from about 4 to 8 μm, and the number of MT per cross-section increased at a similar rate. Over this same time, axonal length increased by a factor of 3.5 as the result of body growth. Somewhat surprisingly the spacing between adjacent MT minus ends, a measure of MT density, remained constant. As body growth causes axons to lengthen by stretching (Smith, 2009; O’Toole and Miller, 2011; Loverde and Pfister, 2015) it is tempting to speculate that as axons stretch, the spacing between the minus ends of MTs already in the axon increases, and new MTs are added in the gaps. Since the density of MT minus ends remains constant through development, the increase in the number of MTs per cross-section may be accounted for by the increase in MT length.

These data complement, but differ somewhat from earlier work using electron microscopy in vertebrates. In hippocampal neurons during the process when a minor stage 3 process (length ≤ 20 μm) transitions into a stage 3 axon (length ≥ 50 μm), MT length is short (∼ 4 μm), and increased axonal length is associated with a rise in the number of MTs and not their average length (Yu and Baas, 1994). In contrast, going from a stage 3 axon to stage 4 axon, there is a large drop in the number of MTs and increase in MT length. Likewise, as axons mature over weeks to months, MT length reaches hundreds of μm and their number declines both *in vitro* and *in vivo* (Bray and Bunge, 1981; Burton, 1987). Keeping in mind that *C. elegans* development occurs over a few days and the study from Shen’s group focused on a period where axons were lengthening (Yogev et al., 2016), a general picture emerges. Early in the process of neurite outgrowth, axonal lengthening is associated with an increase in MT number and an increase in the average or the range of MT lengths. In vertebrates, this is followed by a substantial increase in MT length and drop in MT number (Figure 4). These observations provide a foundation for understanding neurite outgrowth since MT length and number are tightly linked to sliding and the addition of MTs to growing axons.

## MT Transport and Neurite Growth

### MT Sliding in Neuritogenesis

Microtubule sliding is essential for adding and removing MTs from neurites (Lu and Gelfand, 2017; Rao and Baas, 2018). It involves the rapid transport of short polymers (less than 10 μm long) by kinesin-1 and dynein. Kinesin-1 based sliding drives the initial growth of a neurite from the cell body in a process called neurite initiation (Lu et al., 2013). The mechanism involves the coupling of anti-parallel MTs via the motor domain and a second MT binding domain located close to the C-terminus (Winding et al., 2016). As kinesin-1 walks toward the plus end of one MT, it slides the two MTs apart. While sliding is critical for initiation, its involvement in growth once axons reach a length greater than 10–50 μm is less clear; it drops by 50 fold as axons extend over the course of 16 h in *Drosophila* (Lu et al., 2013). One reason for this decline is that kinesin-1 based sliding appears to require that MT have an anti-parallel configuration, but axonal MTs are predominantly parallel (del Castillo et al., 2015b). Somewhat ironically, the generation of parallel arrays of MTs is mediated by dynein based sliding (del Castillo et al., 2015b; Rao and Baas, 2018). As the motor domain of dynein walks along short MTs, it pushes those that point with their plus end toward the cell body out of the axon. In addition to these changes in polarity, the length of MTs increases as axons mature (Figure 4). Because longer MTs have a higher probability of becoming cross-linked with the cytoskeletal array, sliding is also inhibited (Craig et al., 2017). Somewhat surprisingly, the best-characterized class of proteins that suppress MT sliding are the mitotic kinesins: kinesin-5, kinesin-6 (i.e., *Pavarotti*), and kinesin-12 (Lin et al., 2012; Del Castillo et al., 2015a; Rao and Baas, 2018). What they appear to have in common is that they bundle parallel arrays of MTs instead of driving motion. Bringing the ideas of MT polarity, length, and sliding together, a picture emerges that during the initial process of neurite initiation and outgrowth, MTs are short and often have an anti-parallel configuration. Together these allow robust sliding of short MTs that initially increases the number of MTs in the axon. Over time, dynein slides anti-parallel MTs out of the axon, MT length increases, proteins that suppress MT sliding are activated, and MT sliding declines.

Regarding the elongation that follows neuritogenesis, the contribution of MT sliding is less clear. While essentially no rapid sliding has been observed in *Drosophila* axons after this stage (Lu et al., 2013), there are many studies in vertebrate neurons reporting that it occurs in long axons (Wang and Brown, 2002; He et al., 2005); this includes our recent work where we analyzed MT motion using fluorescent speckle microscopy (Athamneh et al., 2017). As our interest is to quantify the contributions that multiple processes make to elongation, we reviewed the data from several key papers and compared it with our findings (Athamneh et al., 2017). This analysis suggested that MT sliding supports the addition of enough MTs to support the extension of the axon at a rate of a few microns per hour (Athamneh et al., 2017), yet axons often extend at much higher rates (i.e., ∼ 25 μm/h in our study). A caveat with this analysis is that a recent paper indicates that much higher levels of MT sliding can occur (Rao et al., 2017). Whether this is due to the use of better markers to track MT motion, the observation of tubulin transported by the endosomal pathway (Chang et al., 1999), or is a function of where and when transport is observed will require a more systematic analysis.

In contrast to MT sliding, analysis of bulk MT transport as we will discuss indicates that it can fully account for the forward movement of MTs needed for growth cone advance. An idea that we favor is that the force generating mechanisms that initially drive robust sliding during neuritogenesis are used to power bulk advance in growing axons (Roossien et al., 2014; Kahn and Baas, 2016). An essential future direction will be to extend the groundbreaking work on MT sliding to test the biophysical contributions of the mitotic kinesins, kinesin-1, and dynein in the mechanics of axonal elongation.

### Axons Elongate Through Bulk Transport of MTs

Whether MTs move in bulk or are stationary relative to the substrate has been debated for roughly 40 years (Hoffman and Lasek, 1975; Bamburg et al., 1986; Baas, 1997; Hirokawa et al., 1997; O’Toole et al., 2008a). It stirs passions because it underlies our fundamental understanding of how axons elongate. While some of the earlier MT labeling studies showed clear evidence of MT translocation during axonal elongation (Reinsch et al., 1991), others did not (Lim et al., 1990; Okabe and Hirokawa, 1990). Roughly a decade ago, while investigating fast mitochondrial transport (Miller and Sheetz, 2004), one of the co-authors noted that ‘docked’ mitochondria, i.e., stably bound to MTs, actin filaments, and neurofilaments (Sheng and Cai, 2012), were not stationary relative to the substrate. Using kymographs to track their motion over long periods indicated that they moved in a coherent manner consistent with bulk MT flow (Miller and Sheetz, 2006). To test if mitochondria move through or with the axon, the motion of beads bound to the outside of the axon and axonal branch points were tracked (Lamoureux et al., 2010). As beads and branch points moved in a pattern similar to mitochondria, this suggested that the cytoskeletal framework moved forward as a whole. In parallel, studies in *Aplysia* growth cones indicated that MTs also undergo forward translocation in the growth cone central domain during adhesion-mediated neurite advance (Lee and Suter, 2008; Schaefer et al., 2008). Quite strikingly, while the prevalent model was that MT assembly drove elongation, 80% of the advance of MTs was accounted for by translocation. To test if bulk motion was an artifact of tissue culture, docked mitochondria were followed *in vivo* in intact *Drosophila* embryos. As seen *in vitro*, they advanced in tandem with the growth cone (Roossien et al., 2013). While these different approaches in several model systems suggested that bulk MT motion accounts for elongation, they were in part indirect: docked mitochondria are not MTs, and the advance of MTs in response to a bead attached to the surface of a growth cone does not reflect axonal elongation. The critical experiment to directly track the bulk motion of MTs in freely growing neurites using fluorescent speckle microscopy was needed to test that hypothesis that bulk MT motion accounted for axonal elongation.

To address this question, the co-authors recently collaborated to track the motion of docked mitochondria, MTs, and the overall motion of cytoplasmic material in both rapidly growing chick and *Aplysia* neurites (Athamneh et al., 2017). This study intended to answer several critical questions: (1) What is the relationship between MT translocation, MT assembly, and neurite elongation; (2) Is the fundamental process of axonal elongation conserved between species? Moreover, (3) is the motion of docked mitochondria a reliable marker for the motion of MTs? The clear answers from these experiments were that MTs advance in bulk at the same average rate as growth cones, the process of elongation is highly conserved, and the bulk motion of docked mitochondria and MTs is highly correlated.

### Does MT Advance Drive Axonal Elongation?

**FIGURE 5:**
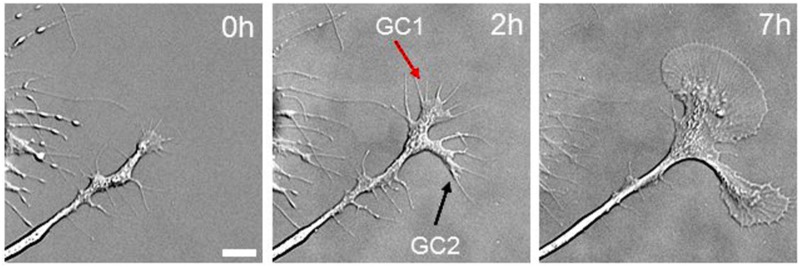
Mass addition to the growth cone does not drive axonal elongation. Differential interference contrast images of *Aplysia* bag cell neuronal growth cone immediately after cell plating (left), 2 h later (middle), and 7 h later (right). Scale bar: 10 mμm. Reprinted from Ren and Suter (2016) with permission from Hindawi.

The strong correlation between bulk MT transport and axonal elongation raises the question of whether bulk MT transport drives elongation. One of the authors’ recent *in vitro* study in *Aplysia* growth cones provides insights into this problem and addresses the question of why *Aplysia* growth cones are much larger than those of other species (Ren and Suter, 2016). When *Aplysia* neurons are plated on poly-L-lysine-coated coverslips, they initially extend several short neurites that have relatively small growth cones, which rapidly expand to the well-known large fan-shaped growth cones of 100 μm in diameter (Figure 5). After the initial process of extension, the leading edge of the growth cone slowly advances at a rate of 1–5 μm/h, but the neck of the growth cone stays in position. At this point, growth cone advance mainly reflects an increase in the size of the growth cone rather than translocation. Over time, the width of the axon is relatively constant, but the growth cone becomes dramatically larger (Figure 5). Based on the change of growth cone area, a large amount of cytoskeletal and organelle mass is being added to the growth cone with only little net advance. Although MTs were not imaged in this study, it has previously been shown that both MT assembly and forward translocation occur in these large growth cones that exhibit minimal net advance (Schaefer et al., 2002; Lee and Suter, 2008). This phenomenon is not unique to *Aplysia*. When Xenopus growth cones rapidly advance, MTs translocate forward, there are relatively few MTs in the growth cone, and they tend to be splayed. When growth cones pause spontaneously, bulk MTs advance continues, and this is paired with MT looping and accumulation (Tanaka and Kirschner, 1991; Tanaka et al., 1995). Likewise, in chick sensory neurons when growth cones pause either spontaneously or when the growth cone is held in position using a towing needle, a dramatic increase in the number of mitochondria in the growth cone occurs as the result of bulk translocation (Miller and Sheetz, 2006; O’Toole et al., 2015). The observation that bulk transport occurs during growth cone pauses suggests it does not drive elongation. In doing so, these observations raise the question, ‘What is the relationship between bulk transport and elongation?’

In our fluorescent speckle microscopy analysis of MT motion, we found that on average there is a one to one correlation between bulk MT transport and the average rate of growth cone advance (Athamneh et al., 2017). Nonetheless, we do not interpret this correlation as indicating that bulk transport causes elongation. Looking at the regression of axonal MT velocity and growth cone velocity in both chick and *Aplysia* neurons (Athamneh et al., 2017), the rates of bulk advance only rarely matches the rate of growth cone advance over 10-min intervals. For example, there are cases where the growth cone is stationary, but bulk advance occurs at 25 μm/h; likewise, there are equal numbers of cases where the converse occurs. As growth cone velocity is characterized by alternating periods of retractions, pauses, and advances, a picture emerges that growth cones go through cycles, where they rapidly advance, deplete the material in the growth cone and relieve compression on MTs. When pauses occur, material accumulates, and MT compression occurs (Rauch et al., 2013). This suggests that the primary role of bulk transport is delivering material to the growth cone. While this is likely a critical step in the cycle needed for sustained outgrowth, the process of growth cone advance over short timescales appears to be more tightly linked to whether the transition zone and peripheral domain move forward. In the next section, we develop a biophysical understanding of elongation that considers the interplay between forces generated by actin, MTs, and the plasma membrane that control this process.

## Force and Motion

### The Biophysical Properties of Neurons

**FIGURE 6:**
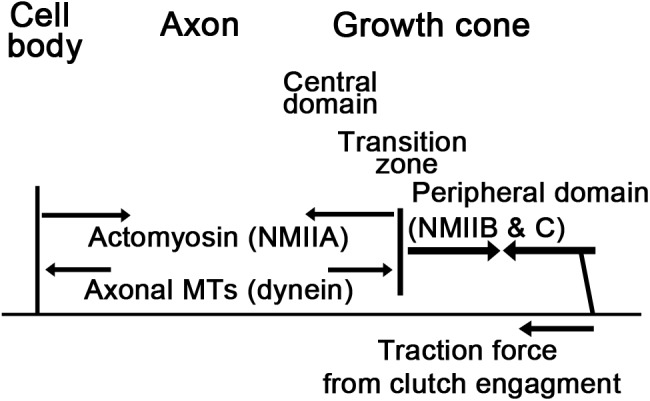
Neuronal force balance. Strong contractile forces by NMIIB and NMIIC at the leading edge pull the transition zone and central domain forward. These forces are countered by NMIIA in the axon and assisted by extensile forces generated through dynein mediated sliding of MTs and MT assembly. Axonal elongation occurs when the traction forces that pull the transition zone forward are higher than the net contractile forces generated in the axon. Arrows represent forces.

Understanding neurite outgrowth requires a detailed knowledge of the biophysical properties of neurons (Suter and Miller, 2011; Franze et al., 2013). Neurons from different species significantly differ in their biophysical properties and levels of force production, respectively (Spedden and Staii, 2013; Athamneh and Suter, 2015). Several approaches have been developed to measure elasticity, viscosity, and force generation in neurons. Particularly valuable tools have been glass microneedles (Bray, 1984; Lamoureux et al., 1989; Suter et al., 1998; Bernal et al., 2007; Athamneh et al., 2015; O’Toole et al., 2015) and more recently microelectromechanical (MEMs)-based force sensors (Siechen et al., 2009; Rajagopalan and Saif, 2011). Typically their bending constants or stiffness values are determined first (Lamoureux et al., 2011), and then forces are measured and applied by optically measuring the amount of bending and controlling their position. Complementing these are innovative approaches using atomic force microscopy (Xiong et al., 2009; Betz et al., 2011; Athamneh et al., 2015), magnetic tweezers (Kilinc et al., 2014; Grevesse et al., 2015), fluid flow (Bernal et al., 2010), vibration of the axon (Garate et al., 2018), FRET-based fluorescent force sensors (Krieg et al., 2014), traction force microscopy (Chan and Odde, 2008; Koch et al., 2012), and laser tweezers (Cojoc et al., 2007; Shahapure et al., 2010; Amin et al., 2013). With these techniques, it is well established that axons behave as solid-like materials in response to transient forces applied for less than 10 s (Bernal et al., 2007; Betz et al., 2011), yet as fluids in response to constant forces applied for tens of minutes (Zheng et al., 1991; O’Toole et al., 2008a, 2015). Depending on the type of neuron, axons generate a net tension between 0.5 and 4 nN (Athamneh and Suter, 2015) that requires actin, ATP expenditure, and myosin activity (Dennerll et al., 1988; Lamoureux et al., 1989; Bernal et al., 2007; Tofangchi et al., 2016). Balancing contraction by actomyosin, MTs bear compressive forces which may be generated in part by dynein (Dennerll et al., 1988; Roossien et al., 2014) and MT assembly (Figure 6). While detailed measurements are essential for quantifying neuronal mechanics, it is equally important to have a theoretical framework to place these observations into context. For example, if one views neurons as solid-like materials, while constant forces generate ‘pre-stress,’ they do not lead to continuous motion needed to account for bulk MT motion. Likewise, simple fluid-like models of neurons fail to capture the solid-like behavior of neurons needed to understand injury arising from a traumatic impact. Reconciling the paradoxical behaviors of neurons requires the adoption of more sophisticated models that are at the cutting edge of soft matter physics.

### Are Neurons Active Fluids?

The mechanical behavior of cells is controlled by structures and processes that are far from thermodynamic equilibrium (Prost et al., 2015). While the study of such systems once was beyond the bounds of conventional physics, a new field has emerged called active gel or active matter physics that considers the influence of internal force generation on mechanics (Marchetti et al., 2013). The unique properties of active matter systems are that they spontaneously generate motion and re-organization of the material. While our focus is on the neuronal cytoskeleton, the theory developed in this field is relevant to flocking, vibrating granules, and microorganisms in suspension (Kumar et al., 2014; Lopez et al., 2015). The mathematics that describes active fluids are complex as they are typically expressed using tensor calculus and consider changes in local orientation, thermodynamics, stress, strain, and motion over time and space (Marchetti et al., 2013; Prost et al., 2015). To explain active matter modeling of the cytoskeleton simply in words, the working assumptions are that it is a Maxwell fluid, similar to Silly Putty, which contains ‘force dipoles’ (i.e., motors that can pull material together and/or push it apart) (Callan-Jones and Julicher, 2011; Prost et al., 2015). When the cytoskeletal elements are aligned, long-distance gradients in stress and motion occur over space and time. Building on this, one of the co-authors have described axons as active Maxwell fluids with the equation (Eq. 1),

(1) $$\dot{\varepsilon }=\frac{\sigma_{\mathrm{Ext}}+\sigma_{\mathrm{Int}}}{\eta }+\frac{{\dot{\sigma }}_{\mathrm{Ext}}+{\dot{\sigma }}_{\mathrm{Int}}}{E};\eta =E\tau$$

where the strain rate (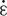) is equal to the constant stress (σ = force / area) arising from both external and external sources divided by viscosity (η), plus the change in stress over time (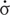) divided by the Young’s modulus (*E*) (de Rooij et al., 2017). Importantly, viscosity and elasticity are related by a time constant (τ) which is in the range of seconds to minutes in cells (Julicher et al., 2007; Betz et al., 2011; Purohit, 2015). Over periods significantly shorter than τ cells behave like solids, while at longer times they act as fluids (O’Toole et al., 2015).

A concrete way to imagine the cytoskeleton as an active Maxwell fluid is to consider it as a system of rods that are highly cross-linked by dynamic springs (de Rooij et al., 2017). τ is related to the *K*_on_ and *K*_off_ rates that describe the binding and unbinding of the cross-links, *E* is related to the spring constants of the cross-linkers and rods, and viscosity is an emergent property. By emergent property, we mean that the system is at a given time point a solid with a modulus of *E*, but flows as a fluid because the cross-links are dynamic as described by τ. When the system is under stress, motion occurs because the unbinding and rebinding of cross-linkers dissipates energy and gives rise to ‘permanent’ deformation. In turn, the generation of internal forces by motors occurs by linking ATP consumption to the shortening of cross-linked springs. In essence, when a motor undergoes a power stroke, it is converted from a long to a short spring (de Rooij et al., 2017). This length change generates tension, which pulls materials together.

The utility of a model is based on how well it reflects observed behavior. This raises the question ‘Do axons behave as active Maxwell fluids?’ There are several lines of evidence that suggest so. Bernal’s group (Bernal et al., 2007) demonstrated that over short times (<10 s), axons behave like solids and actively generate forces. In contrast over minutes to hours, there is a steady rate of actin retrograde flow in the growth cone (Betz et al., 2011) coupled with a continuous bulk forward advance of material along the axon (O’Toole et al., 2015; Athamneh et al., 2017). The study by Betz et al. (2011) is notable in that it was the first to apply principles of active fluids to understand growth cone mechanics. We also would like to highlight the application of these principles for modeling the retrograde flow of *Aplysia* growth cones (Craig et al., 2012) and bulk motion during neurite outgrowth (O’Toole et al., 2008a; Recho et al., 2016).

While there is broad agreement that actin in the growth cone is an active fluid (Betz et al., 2011; Craig et al., 2012; Recho et al., 2016), whether axonal MTs are as well has a more complicated history. In the first well-articulated biophysical model for axonal elongation (Dennerll et al., 1989), axonal MTs where modeled as a viscoelastic solid (i.e., a spring in series with a spring and dashpot in parallel), capped with a fluid-like dashpot at the growth cone. While this system as a whole is a viscoelastic fluid, the axon was considered a viscoelastic solid. The justification for this arose from suggestions that MTs are stationary along the axon, while new MTs are dynamically assembled and disassembled in response to forces at the growth cone (Bamburg et al., 1986; Heidemann, 1990). While there have been numerous models that treat axons as solids, typically referencing (Dennerll et al., 1989), more recent studies suggest that axons internally stretch like a fluid (O’Toole et al., 2008a, 2015). This idea is supported by the fact that mixtures of MTs and kinesin behave as active fluids (Wu et al., 2017), MTs slide as single filaments (Lu and Gelfand, 2017; Rao and Baas, 2018), axons take up slack (Tofangchi et al., 2016), and growth cone retracts back to the cell body in a fluid-like manner when adhesion to the substrate is disrupted (O’Toole et al., 2015). To put this shifting view of axonal mechanics in perspective, while two of the founders of the field of neuronal mechanics, Dennis Bray and Steven Heidemann, once favored the idea of a solid-like axon, they now support the view they are fluid-like structures (Heidemann and Bray, 2015). In summary, the available evidence strongly indicates that axons and the MT arrays contained within them act as fluids during the process of neurite outgrowth. By adopting a model of neurons as active Maxwell fluids, the study of axonal elongation can advance to a state where the relative contributions of diverse mechanisms, over a broad range of time scales, can be considered and related in a unified model (de Rooij et al., 2017, 2018; de Rooij and Kuhl, 2018).

### Forces in Series

A major challenge in cell mechanics is relating specific force measurements, typically made by probing the outside of cells, with the profile of subcellular force generation within a cell (Park et al., 2010; Canovic et al., 2014). Whereas it is intuitive that parallel forces sum (e.g., consider two people pushing a box), how forces combine in series as is the case of an axon attached to a growth cone, is rarely discussed. It is well documented that neurons generate traction forces on the order of 1 nN, and it is typically assumed that growth cones are the primary site of force generation. However, does this mean that growth cones generate 1 nN of force? To better understand this, one the co-authors systemically considered the problem of how forces interact in series (O’Toole et al., 2015). Without going into the details of its derivation, the equation that describes the relationship between net force and the forces generated in the axon and growth cone is given by Eq. 2.

(2) $$F_{\mathrm{Net}}=F_{\mathrm{GC}}\frac{\eta_{\mathrm{Axon}}}{\eta_{\mathrm{GC}}+\eta_{\mathrm{Axon}}}+F_{\mathrm{Axon}}\frac{\eta_{\mathrm{GC}}}{\eta_{\mathrm{GC}}+\eta_{\mathrm{Axon}}}.$$

Here, we have a model where the neuron has two compartments that can have different viscosities (η) and levels of internal force generation (*F*). On one end, the axon is attached to a fixed point, and on the other, the growth cone is affixed to a means to measure net force. If we assume for the moment that the viscosity in both regions is the same, the net force is equal to the average force. For example, if forces were only generated in the growth cone, the net force would be equal to half of that. An intuitive explanation for why this occurs is that when no forces are generated in the axon, contraction occurs in the growth cone. Since this motion is coupled with the dissipation of forces through viscosity, the force measured externally is reduced. This equation implies that the measured net force provides little information about the site and magnitude of internal force generation; just because a growth cone pulls on the substrate, does not mean it is the primary site of force generation.

The theory outlined in Eq. 2 lead us to ask where are forces generated in neurons and what is their magnitude. In thinking about neurons are active fluids, it became clear that when the local internal forces are equal to the external forces, the local region neither expands or contracts (O’Toole et al., 2015). Using towing needles attached to the growth cone to systematically vary the external force while monitoring subcellular strain rate by tracking docked mitochondria, we found the contractile force across the central domain to be 2 nN, while along the axon it was 0.6 nN. In turn, the average net force for the chick sensory neuron as a whole was 1.3 nN. Because the net force was close to the average of the forces in the two regions, this suggests the viscosity of the rear of the growth cone (which contains MTs in the central domain surrounded by actin arcs) is similar to the viscosity of the axon (which is composed of axonal MTs surrounded by an actin cortex) (Figure 2B). In addition, it makes clear that the traction forces measured at the growth cone (Chan and Odde, 2008; Koch et al., 2012) are a complex function of the subcellular profile of force generation (Figure 6).

The second implication of this model is that net traction forces generated by neurons are likely independent of the whether axons elongate or not. Combining Eqs. 1 and 2, the rate the axon lengthens is determined by the difference in forces generated in the growth cone and axon, divided by the viscosity of the axon. While fast elongation could be paired with high levels of force generation in the growth cone as the result of clutch engagement, it could also result from low levels of force generation in the axon (Figure 2). The study by Hyland looked carefully at the correlation between net traction force and the rate of elongation (Hyland et al., 2014). They found, in essence, no strong correlation and noted that the highest traction forces were often generated by the slowest growing neurons. As we will develop below, we think the reason for this is that Rho selectively leads to the activation of NMIIA along the axon, which generates a contractile force that opposes growth cone advance (Figures 2C, 6). This suggests that it is more critical to consider the subcellular pattern of force generation rather than the total force. In discussing these results, Hyland et al. (2014) pointed out that it was paradoxical that there was no strong correlation between traction forces and outgrowth rate, yet there is a strong correlation between externally applied forces and elongation. Based on one of the author’s recent modeling study (de Rooij et al., 2018), we think a possible explanation is that external forces directly cause the axon to stretch or contract as a compartment. In contrast, in neuronal cultures traction force arises from the balance of forces between the axon and growth cone. Thus, higher traction forces do not necessarily equate to faster elongation (Figure 6).

In conclusion, a key determinate of whether axons elongate, stall or retract appears to be the gradient in the force profile from the axon across the growth cone (Figure 2C). When the levels of force generation in the axon and growth cone are similar, high traction forces will be produced, but the material will not flow forward. In turn, if the level of contractile forces is higher in the axon than in the growth cone, retraction occurs. Only when the levels of force generation are higher in the growth cone than the axon, material flows forward (Figure 6). A secondary and related idea is that viscosity controls the rate of material flow (O’Toole et al., 2008a). In general, changes that decrease viscosity without altering forces will lead to faster rates of elongation and retraction, whereas inhibiting the dynamics of cytoskeletal elements or increasing the number or the stability of cross-linkers will have the reverse effect (de Rooij et al., 2017, 2018).

### Axons Elongate at the Rate of Transition Zone Advance

With this background on neurons as active materials in mind, we can now return to the question of the relationship between bulk MT transport and growth cone advance. Combining estimates of subcellular forces generation (O’Toole et al., 2015) with a detailed analysis of MT motion in growing neurites (Athamneh et al., 2017) provides a comprehensive picture of subcellular force generation and motion (Figures 2C,E). Far from the growth cone (>200 μm), MTs are stationary relative to the substrate (Miller and Sheetz, 2006). In the growth cone, MT translocation velocity rises to an average rate of ∼ 25 μm/h (Athamneh et al., 2017). This gradient in velocity reflects the stretching of the axon. It is caused by the difference in contractile forces between the axon and the traction forces generated in the growth cone and shaped by the frictional interactions between the axon and substrate (O’Toole et al., 2008a; Figure 2D). Across the growth cone, MT velocity drops sharply, to a large negative value of ∼−125 μm/h over a region of ∼ 5–10 μm in the peripheral domain (Athamneh et al., 2017). The switch from slow bulk anterograde motion to rapid retrograde flow occurs at the transition zone when MTs enter into the peripheral domain and become coupled with retrograde actin flow (Schaefer et al., 2002, 2008; Lee and Suter, 2008). As the growth cone advances over time, this profile moves forward in space at a rate roughly equal to a peak rate of MT advance in the growth cone (Athamneh et al., 2017).

While, we measure velocity in one dimension, axons are three-dimensional, and volume is conserved during rapid stretching (Fan et al., 2017). If we ignore for the moment the delivery of material through fast and slow axonal transport, changes in velocity along the axis of the axon are paired with alterations in diameter. In regions where the velocity gradient is positive (i.e., it increases with distance from the cell body) ‘stretching’ occurs and diameter decreases, likewise in the growth cone where the velocity gradient is negative diameter increases. Mechanically, this explains in part why growth cones are wide, and axons are thin. Strikingly, if new materials were not added to axons, they would rapidly thin behind the growth cone (O’Toole et al., 2008a).

Nonetheless, neurons are not passive materials: the subcellular assembly and disassembly of MTs are tightly controlled. Because axonal diameter remains relatively constant during elongation (Bray, 1984), fast axonal transport of organelles, slow axonal transport of cytoskeletal elements, and net MT assembly are needed to maintain axonal diameter (O’Toole et al., 2008b). In support of this, the flux associated with transport declines along the axon, which adds mass (Miller and Samuels, 1997; O’Toole and Miller, 2011). Paired with this, analysis of end-binding (EB)1/3 protein comets and markers for MT dynamics indicates that MT assembly occurs preferentially in the distal axon, where stretching is most prominent (Baas and Black, 1990; Cammarata et al., 2016; Qiang et al., 2018). Thus, while our recent quantitative analysis of MT motion indicates that axons do not advance by the assembly of MTs at the tip of the central domain (Athamneh et al., 2017), analysis of the velocity gradients suggests robust assembly is needed in the distal axon to prevent thinning. The observation of robust MT assembly in the distal axon raises the question, ‘why doesn’t MT assembly at tip drive elongation.’ One possibility is that when MTs extend into the peripheral domain, they come under high stress, which bends and breaks the plus ends off (Schaefer et al., 2002, 2008; Lee and Suter, 2008; Rauch et al., 2013). In turn, MT disassembly may occur (Tang-Schomer et al., 2010). Thus, the same MTs that could add mass at the tip of the central domain are the ones most likely to depolymerize.

Based on these considerations, we propose that the transition point, where the velocity gradient shifts from positive to negative (Figure 2E), regulates the shape and size of the growth cone (Figure 5). Behind this point, axonal thinning occurs because of stretching. In front of this point, thickening occurs through contraction. When the growth cone pauses, this transition point shifts back toward the contractile node in the neck of the growth cone, which leads to the widening of the central domain because contraction pushes material outwards. In contrast, during rapid elongation, this point shifts forward toward the actin-rich region in front of the growth cone, which causes the central domain to stretch and decrease in width. As the transition zone and actin arcs surrounding the sides of the growth cone are critical for creating this velocity gradient, the question of what controls its advance becomes critical for understanding elongation. As we develop next, the net forces generated by multiple sources including membrane tension, MTs assembly, axonal contraction, actin retrograde flow, and clutch engagement are integrated into a net force vector across the transition zone that controls its advance (Figures 2, 6). In terms of the biology, a critical question is the relative force contributions of each of these mechanisms.

### Does MT Assembly Create a Pushing Force That Drives Elongation?

The observed correlation between bulk MT advance and elongation (Athamneh et al., 2017) challenges the idea that new tubulin addition at the tip is the main driving force for neurite elongation. To determine if MT assembly modulates bulk transport, we disrupted it and tracked bulk motion (Athamneh et al., 2017). Strikingly, this blocked forward advance, lead to retraction of material in the distal axon, and increased tension by roughly 60%. Collectively this suggests that normal MT assembly is needed for elongation because when it is disrupted, tension rises (presumably along the axon) that pulls material rearward. While models of axonal elongation have suggested that MT assembly generates a pushing force that drives elongation (Buxbaum and Heidemann, 1992; Recho et al., 2016), we suspect that the large compressive forces created by MTs are primarily generated as the result of MT sliding by motors and coupling between MTs and actin retrograde flow via cross-linkers. For MT assembly to generate a significant pushing force, it must be stalled against a barrier (Dogterom and Yurke, 1997). In agreement with this, EB3-GFP comets do not stall along the axon (Stepanova et al., 2010), as they would if they were pushing against a barrier. In addition, as the stall force of a MT is ∼ 5 pN (Dogterom and Yurke, 1997) and the numbers of MTs in a typical growth cone are in the range of 5 - 20, the force generated by assembly is theoretically small compared to the net forces generated by NMII and dynein that act on MTs (Koch et al., 2012; Roossien et al., 2014). If we exclude the idea that MT assembly generates large extensile forces, why does tension rise so dramatically when assembly is blocked? Here, we see two untested but logical possibilities. The first is that disruption of MT assembly interferes with the ability of dynein to generate large extensile forces which have been measured to be at least 400 pN in sensory neurons (Roossien et al., 2014). As dynein is a +tip protein (Duellberg et al., 2014) and drugs that target MT dynamics lead to disassociation of +tip proteins from MTs (Morrison et al., 1998), disruption of MT assembly could dramatically reduce the ability of dynein to generate extensile forces on MT arrays (de Rooij et al., 2017). Secondly, disruption of MT assembly could lead to the activation of NMII along the axon via the GEF-H1 – Rho signaling pathway (Chang et al., 2008; Takano et al., 2017; de Rooij and Kuhl, 2018). While the idea that MT assembly generates a pushing force that drives elongation has been an attractive one, we believe that there are plausible alternative hypotheses, which in our eyes need to be tested.

### Is the Growth Cone a Battering Ram?

Cajal initially compared the growth cone with “a living battering ram, soft and flexible, which advances, pushing aside mechanically the obstacles which it finds in its path” (Ramón y Cajal, 1995). This evokes an impression that it pushes forward with large forces. Consistent with this idea actin filaments polymerize at the leading edge (Forscher and Smith, 1988), and their assembly generates a force of 5–10 pN per filament *in vitro* (Greene et al., 2009; Figure 2). As there are 100–200 actin filaments per micron at the leading edge of cells (Koestler et al., 2008), a 1 μm region could generate as much as ∼ 1 nN/μm. Furthermore, in non-neuronal cells, the forward pushing force of the lamellipodia has been directly measured using atomic force microscopy to have a stall force of roughly 0.3 nN/μm (Prass et al., 2006). Similarly, using a more refined analysis, the pushing force associated with actin assembly in the lamellipodia of *Aplysia* neurons has been estimated theoretically to be ∼ 100 pN/μm (Craig et al., 2012). As the length of the leading lamellipodial edge in a typical chick sensory growth cone is ∼ 5–10 μm, these growth cones could theoretically push forward with 0.5–1 nN of force. This is similar to the pulling or traction force, ∼ 1.5 nN, of these growth cones (Athamneh and Suter, 2015; O’Toole et al., 2015).

In contrast to theory, experimental data suggest that the forward pushing force of actin assembly in growth cones is extraordinarily small. When the pushing force of growth cones was measured directly in sensory or hippocampal neurons, individual filopodia pushed forward with ∼ 1–5 pN and the lamellipodia with ∼ 10–20 pN/μm (Cojoc et al., 2007; Amin et al., 2013). If we assume that the length of the leading edge of a growth cone is 10 μm, the net force would be 100 pN; roughly 10 times less than the traction forces (O’Toole et al., 2015). One possibility to explain these small forces is that membrane tension is very high and most of the forces associated with actin assembly are directed to pushing it forward. Yet, the in-plane membrane tension in chick sensory and *C. elegans* neurons are 3 pN/μm and ∼ 12 pN/μm, respectively (Hochmuth et al., 1996; Krieg et al., 2014). This suggests that the small measured pushing force of growth cones does not arise because large forces that counteract each other. Instead, membrane tension and the force of actin polymerization are both small and balanced. This is consistent with the idea that membrane tension may be determined by the tension at which actin polymerization underneath the membrane is mechanically stalled (Sens and Plastino, 2015). Regarding growth cone behavior, the close balance between these forces has also been suggested to account for the probabilistic anterograde and retrograde motion of the growth cone (Shahapure et al., 2010). While membrane addition is critical for the process of axonal elongation and blocking membrane addition halts elongation (Quiroga et al., 2018), membrane tension does not appear to be significantly limit the assembly of actin or the advance of the growth cone through large forces.

### Substrate-Cytoskeletal Coupling

In addition to pushing forward, most types of growth cones generate pulling or traction forces in the range of 0.5 to 3 nN (Athamneh and Suter, 2015). Neuronal cell lines and central nervous system neurons generate forces at the lower end of the range, whereas peripheral nervous system neurons build up moderate forces. An exception is the enormous growth cones of *Aplysia* neurons, which can develop traction forces up to 100 nN (Athamneh et al., 2015). Strikingly, single filopodia in chick sensory neurons can pull with a force of ∼ 1 nN (Heidemann et al., 1990; Bridgman et al., 2001) suggesting that forces in the growth cone can be highly directed. Nonetheless more typically, traction force analysis indicates that forces are distributed over the growth cones, with a few peaks of high intensity (Koch et al., 2012; Hyland et al., 2014; Figure 2D). How do retrograde traction forces in the growth cone develop, and what is their function?

The substrate-cytoskeletal coupling model explains the generation of traction forces by proposing that point contacts link actin undergoing retrograde flow to the extracellular matrix (Suter et al., 1998; Bard et al., 2008; Shimada et al., 2008; Nichol et al., 2016). Engagement of the clutch through linkages between the actin cytoskeleton and the adhesion receptors, through proteins such as talin, vinculin, shootin1, and cortactin (Toriyama et al., 2013; Gomez and Letourneau, 2014; Kubo et al., 2015) increases traction forces and slows flow (Figures 2, 6). Relatively little is known how guidance cues and signaling affect force production in growth cones and neurites. The few studies that have published in this area focused on how signaling regulates clutch formation and thereby traction force. The Inagaki group has shown that netrin-1 causes Pak1-mediated shootin1 phosphorylation to regulate substrate-cytoskeletal coupling and traction force production (Toriyama et al., 2013). Phosphorylation is probably the most common post-translational protein modification associated with force production. Along these lines, Suter and Forscher have shown that strong coupling between the cell adhesion molecule apCAM and actin cytoskeleton in *Aplysia* growth cones depends on Src-mediated tyrosine-phosphorylation (Suter and Forscher, 2001).

How substrate-cytoskeletal coupling translates into growth cone advance is not fully understood. A classic interpretation of the clutch hypothesis is that it reduces the rate of retrograde actin flow, and increases the rate of growth cone advance (Mitchison and Kirschner, 1988; Suter et al., 1998; Suter and Forscher, 2000). The reduced actin flow consequently represents a reduced barrier to MT assembly into the peripheral domain (Hur et al., 2011; Cammarata et al., 2016; Blanquie and Bradke, 2018). Whereas there have been several reported examples where the growth cone transition from one substrate to another resulted in an inverse relationship between actin flow and growth cone advance (Lin and Forscher, 1995; Suter et al., 1998; Nichol et al., 2016), slower rates of retrograde flow are not always linked to faster elongation. For example, when *Aplysia* growth cones are treated with serotonin (i.e., 5-HT) both retrograde flow and elongation increase (Zhang et al., 2012). Disruption of NMII with blebbistatin reduces traction forces, retrograde flow, and elongation when neurons are grown on laminin (Medeiros et al., 2006; Ketschek et al., 2007; Koch et al., 2012). Inhibition of actin disassembly reduces retrograde flow, traction forces and elongation (Gallo et al., 2002; Van Goor et al., 2012; Hyland et al., 2014). Finally, when we examined the correlation between retrograde flow and axonal elongation, flow rates increased as the rate of elongation rose (Athamneh et al., 2017). In light of these findings, we believe that the primary function of the clutch is not to reduce actin flow, but rather to increase the tension between adhesions and the transition zone (Figure 2). When this causes the net force vector over the transition zone to be positive, the transition zone advances and MTs flow forward in bulk.

A general prediction of this model is that higher rates of elongation should be paired with higher traction forces. In support of this, it is well established that traction forces and elongation rise when the clutch is engaged (Suter et al., 1998; Athamneh et al., 2015; Kubo et al., 2015). Nonetheless, a careful analysis of growth rates and traction forces in freely growing neurons indicates forces and growth rate are not correlated (Hyland et al., 2014). As we indicated above and will develop in the next sections, the growth cone is not the only source of force generation in neurons. In addition, contractile forces are generated along the axon (O’Toole et al., 2015; Tofangchi et al., 2016) that could oppose the advance of the transition zone (Figures 2, 6). Furthermore, extensile force generation by the MT cytoskeletal is posed to decrease traction forces and boast elongation (Roossien et al., 2014). This suggests that axonal elongation is not controlled by a single process, but rather how multiple mechanisms interact (Figure 2). Through the next sections of the paper, we expand on the ideas introduced here to develop a more detailed understanding of the contribution of NMII, actin turnover, and actin-MT coupling in elongation.

### Force Generation by Non-muscle Myosin II in the Growth Cone

Non-muscle myosin II has a central role in modulating axonal elongation and neuronal mechanics. It acts downstream of the major classes of guidance cues and signaling pathways including Slit, Netrin-1, Semaphorin-3A, Ephrin-A5, Rho and ROCK (Wahl et al., 2000; Wylie and Chantler, 2003; Gallo, 2006; Brown et al., 2009; Murray et al., 2010). Consistent with this role, it produces the majority of traction forces generated by neurons (Bridgman et al., 2001; Koch et al., 2012). Strikingly, while NMII generates large forces, it is not required for axonal elongation *per se*. Treatment of chick sensory neurons with 50 μM blebbistatin, which reduces both NMIIA and NMIIB activity by >95% (Limouze et al., 2004), only decreases the rate of axonal elongation on laminin by 66%, and increases the rate on poly-lysine by ∼ 50% (Ketschek et al., 2007). Furthermore, disruption of NMII only slows retrograde flow by 50% in *Aplysia* growth cones (Medeiros et al., 2006). These observations suggest that while NMII generates large forces, there are other motors and force-generating mechanisms which power outgrowth in its absence. Instead, the primary role of NMII appears to be the modulation of outgrowth downstream of guidance cues.

A key to understanding the complex function of NMII is that there are three isoforms, NMIIA, NMIIB, and NMIIC, all of which are expressed at relatively high levels in the brain and each with specific, yet overlapping functions (Golomb et al., 2004; Wylie and Chantler, 2008; Shutova and Svitkina, 2018). Of note, disruption of NMIIB or NMIIC slows neurite elongation for N2A cells grown on fibronectin, while disruption of NMIIA increases it (Wylie and Chantler, 2001). More generally, NMIIA is recognized as promoting axonal retraction, while NMIIB drives elongation (Bridgman et al., 2001; Kubo et al., 2008; Wang et al., 2017). While all three are found in the growth cone, axon shaft, and cell body, there are variations in their peak levels of localization. NMIIA is found to be most concentrated along the axon shaft and central domain (Rochlin et al., 1995; Bridgman, 2002; Wylie and Chantler, 2008), whereas NMIIB and NMIIC are enriched in the transition zone (Rochlin et al., 1995; Wylie and Chantler, 2008; Figures 2C, 6). In turn, treatment of neurons with Semaphorin 3A, which causes growth cone collapse and retraction, increases the concentration of NMIIA in the axon and shifts NMIIB from the transition zone in front of the central domain to the neck of the growth cone (Gallo, 2006; Brown et al., 2009). Activation of Rho, which preferentially activates NMIIA in neurons (Kubo et al., 2008), drives contraction in the axon but does not affect retrograde flow (Zhang et al., 2003). Thus, a picture emerges that during rapid elongation NMIIB generates contractile forces in the growth cone to promote elongation, while NMIIA generates contractile forces along the axon that oppose it (Figure 6). From these observations, during slow growth or retraction, Rho is activated, which generates contractile forces along the axon mediated primarily by NMIIA. When Rho is inactive, contractile forces in the axon decrease, strong pulling forces generated by NMIIB (and perhaps NMIIC) in front of the central domain are dominant, and rapid elongation ensues.

Whether the disruption of NMII increases or decreases elongation depends on the substrate (Ketschek et al., 2007); when neurons are grown on polyamines, in the presence of growth inhibitory substrates such as CSPGs, or in the presence of low concentrations of laminin, growth is typically slow, and inhibition of NMII increases elongation (Ketschek et al., 2007; Hur et al., 2011). In contrast, growth on high concentrations of laminin is rapid but slowed by inhibition of NMII (Ketschek et al., 2007; Turney et al., 2016). As growth inhibitory substrates activate Rho (Monnier et al., 2003; Geoffroy and Zheng, 2014), these effects can be interpreted in the context of the differential activation of NMIIA along the axon.

To better understand the interplay between the substrate, NMII activity, and growth, Turney et al. (2016) recently investigated the mechanism underlying the promotion of neurite outgrowth by NGF on both fibronectin and laminin in embryonic mouse sensory neurons. They did so by systematically varying NGF concentration, the substrate, and NMII activity. They found NMII disruption blocked the growth promoting effect of NGF, but the mechanism depended on the substrate. On laminin, NGF had little effect on NMII activity as assessed by phosphorylated myosin light chain staining. Instead, it promoted growth by selectively shifting vinculin, which links actin to integrin, to the leading edge of the growth cone. Correlated with this shift, NGF increased traction forces and slowed retrograde flow. In contrast, when neurons were grown on fibronectin, NGF selectively decreased NMIIA activity. As these studies and our recent analysis of bulk MT motion were both conducted in sensory neurons grown on laminin, the possibility exists that the effects of NGF, substrate and NMII activity may be explained through a consideration of how they impact the forward flow of MTs and the motion of the transition zone (Athamneh et al., 2017). In concluding this section, we think it is important to note that our model of how NMII isoforms interact to control elongation is a working hypothesis (Figures 2, 6) and direct biophysical analysis of the role of NMII in sub-cellular force generation and bulk flow is needed to test it.

### The Importance of Actin Disassembly in Neurite Outgrowth

Growth cone advance depends not only on actin assembly and NMII-actin interactions but also on actin disassembly (Figure 2). ADF/cofilins are tightly linked to this process because they promote actin turnover (Bamburg and Bernstein, 2010). They do so by selectively binding to F-actin bound to ADP, severing the filaments, and promoting disassembly at both ends (Wioland et al., 2017). During neurite elongation, inhibition of actin disassembly by either disruption of ADF/cofilin (Endo et al., 2003; Flynn et al., 2012) or with the actin-stabilizing drug jasplakinolide (Gallo et al., 2002; Van Goor et al., 2012), slows retrograde flow and growth cone advance. In contrast, activation of ADF/cofilin downstream of 5-HT increases elongation and retrograde flow (Zhang et al., 2012). The effect of ADF/cofilin in promoting growth has been suggested to occur in part because it creates a space that allows MT advance (Flynn et al., 2012). On the other hand, activation of AC downstream of repulsive cues leads to growth cone collapse paired with a decrease in growth cone F-actin (Hsieh et al., 2006; Piper et al., 2006). If actin filaments were a passive barrier to MT advance, one would predict elongation to increase. Likewise, the observation that both flow and growth slows when actin disassembly is inhibited challenges the hypothesis that that rapid retrograde actin flow is a kinetic barrier to MT advance (Lin and Forscher, 1995). A clue to this complex response comes from the observation that the inhibition of actin disassembly with jasplakinolide decreases traction forces by ∼ 50% (Hyland et al., 2014). As discussed above, viewing elongation as being controlled by force balance, the decrease in traction forces may explain why inhibition of actin disassembly slows elongation.

Why forces decrease when actin disassembly is inhibited is still poorly understood, but may occur because they are shunted toward breaking and compacting actin filaments, instead of pulling the substrate rearward (Medeiros et al., 2006; Craig et al., 2012; Vogel et al., 2013; McFadden et al., 2017). Similarly, loss of growth cone actin as the result of high ADF/cofilin activity may reduce traction forces through loss of linkages with the substrate. It is important to note that the biophysical effects of altering ADF/cofilin on growth cone traction forces are currently unknown. Furthermore, other effects such as a change in viscosity could explain these responses to changing actin disassembly (O’Toole et al., 2008a; de Rooij et al., 2018). Given our field’s poor understanding of the interplay between actin dynamics and neuronal mechanics, we suggest it as a critical topic for deeper investigation.

### MT-Actin Interactions in Neurite Outgrowth

How MTs and actin interact to drive axonal elongation is a difficult question (Coles and Bradke, 2015; Voelzmann et al., 2016). Again, applying ideas developed in physics with rigorous cell biology has the potential to transform our understanding. A growing array of MT plus-end tracking protein, direct cross-linkers, and cross-linking protein complexes have been identified as couplers between actin filaments and MTs (Figure 2). Invariably, disruption of these proteins alters elongation and guidance and leads to disordered MT arrays. An excellent illustration of this is shown in *Drosophila* neurons null for *shot*, the homolog of spectraplakin ACF7, where extensive MT buckling occurs in both the growth cone and along the axon (Alves-Silva et al., 2012). In parallel, theory to model the bending of rods in elastic matrixes and active fluids has been applied to better understand the relationship between MT bucking, forces and the influence of actin (Brangwynne et al., 2006; Kikuchi et al., 2009). When MTs are under compression in isolation, they buckle as rods described by the classic Euler buckling theorem. When MTs are embedded in an active fluid or elastic medium, such as actin filaments, instead of having a single C shaped curve, they assume a wavy S-shaped confirmation (Brangwynne et al., 2006). As the stiffness of the matrix increases, the number of bends (i.e., modes) and the force MTs bear increases, whereas the size of the bends (i.e., their amplitude) decreases (Brangwynne et al., 2006). With knowledge of the stiffness of MTs and the actin meshwork, the compressive force on MTs can be estimated based on their curvature. In neurons and other cells, this is on the order of 100 pN with the caveats that density, orientation, and forces generated by actomyosin strongly impact this estimate (Brangwynne et al., 2006; Rauch et al., 2013).

**FIGURE 7:**
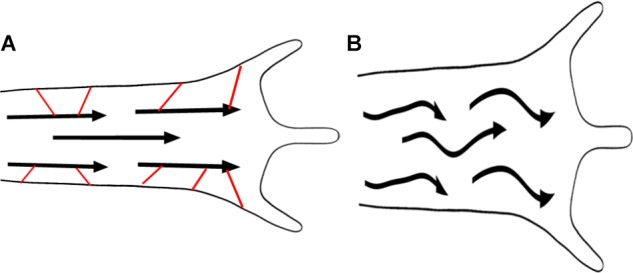
Microtubule/actin coupling promotes elongation. Loss of MT – actin cross-linkers shown in red **(A)** leads to MT buckling **(B)**, shorter axons and thicker growth cones. MTs are represented by arrows.

When actin-MT cross-linkers are disrupted, it seems unlikely that either the compressive forces on MTs are higher or that the actin matrix is directly weakened. Nonetheless, as the coupling between actin filaments and MTs is decreased, it is possible that the ‘effective’ stiffness of the matrix is reduced. From this, the buckling of MTs observed when shot (Alves-Silva et al., 2012), tau (Biswas and Kalil, 2018), and other cross-linkers are disrupted may arise through a decreased physical interaction with the actin network (Figure 7). There are two important implications. The first is that without the stabilizing influence of actin, MTs will bear reduced compressive loads (Brangwynne et al., 2006). The second is that when MTs are disorganized, they will direct forces against the sides of the axon and growth cone (Alves-Silva et al., 2012). Bringing these ideas together provides a physical explanation for the large axonal varicosities filled with disorganized MTs and the widening of the growth cones observed when the actin-MT cross-linking function of tau is disrupted (Biswas and Kalil, 2018). More generally, a reduction in the net forward forces may explain why disorganized MTs are typically associated with reduced rates of axonal elongation. Collectively, these observations shift focus from models that propose elongation is driven by the pushing force of MT or actin assembly in the growth cone toward mechanisms involving MT sliding by motors and crosslinking to actin filaments.

## Conclusion and Outlook

In conclusion, we propose here an integrated cytoskeletal model of neurite outgrowth (Figure 2), that does not pinpoint a single dynamic process as the sole driving force of elongation. We suggest that gradients in force generation and adhesions along the axon and growth cone determine whether axons elongate, retract, or stall. If the growth cone produces stronger traction forces and adhesions than the axon, the net result will be increased neurite growth. The second significant aspect of our model involves the idea that axons are active fluids and that viscosity controls the rate of material flow. In addition to force generation, cross-linkers between different types of filaments affect viscosity and control how quickly flow occurs in response to forces. Since cross-links are lost when filaments undergo disassembly, the dynamics of MTs and actin filaments impacts viscosity. In general, for fast growth to occur, the density of cross-linkers needs to be minimized, and the dynamics of filaments increased. On the other hand, if the density of cross-linkers drops too much, the forces generated by these systems may be less directed (Figure 7).

Viewing neurons as an active fluid leads to a model of elongation that is useful for understanding how growth occurs and further suggests principles for prompting rapid neurite growth for example during regeneration following injury. To promote rapid elongation, one needs to increase net contractile force generation and adhesions in front of the central domain, decrease net contractile force generation and adhesions along the axon, and lower viscosity (Figures 2, 6). As forces, adhesions, and viscosity are influenced by multiple processes; many approaches could lead to fast elongation. What complicates the development of therapies for neurite growth is that any given component is typically involved in several processes that often have opposing effects on elongation. However, without an integrated model, it will be challenging to come up with better approaches to increase neurite growth. We hope that this review will stimulate new developments in this area.

## Author Contributions

All authors listed have made a substantial, direct and intellectual contribution to the work, and approved it for publication.

## Conflict of Interest Statement

The authors declare that the research was conducted in the absence of any commercial or financial relationships that could be construed as a potential conflict of interest.

## Abbreviations

EB: end-binding proteins
MT: microtubule
NGF: nerve growth factor
NMII: non-muscle myosin II.
